# Accurate segmentation of localized fuel cladding chemical interaction layers in SEM micrographs with deep learning method

**DOI:** 10.1038/s41598-025-14927-8

**Published:** 2025-08-07

**Authors:** Liang Zhao, Yachun Wang, Fei Xu

**Affiliations:** Idaho National Laboratory, Idaho Falls, ID 83402 USA

**Keywords:** Metallic fuel, Fuel/Cladding chemical interaction, Scanning electron microscopy, Layer segmentation, Deep learning, Materials science, Mathematics and computing

## Abstract

U-Zr metallic fuels are promising fuel candidates for fast reactor applications. Fuel/cladding chemical interaction (FCCI) is a random, localized, complex interdiffusion phenomenon occurring at the fuel cladding interface under irradiation, thinning the cladding wall. This interaction has been recognized as a limiting factor in deploying metal fuels to achieve higher burnup under steady state operations. The post irradiation examination of Experimental Breeder Reactor II and Fast Flux Test Facility fuel pins with different irradiation conditions have been the primary method for investigating FCCI in metal fuels, utilizing various characterization techniques, including scanning electron microscopy (SEM). This study compared several computer vision and deep learning approaches for the automated segmentation of FCCI layers in SEM micrographs. We deployed and compared state-of-the-art deep learning models for the task of FCCI layer segmentation in SEM micrographs. A deep learning based end-to-end method proved its capability to enable rapid and accurate segmentation of FCCI in as-collected SEM micrographs, making it highly suitable for real-time applications to automate data analysis. The average segmentation mAP achieves a high performance of 90.8% for dozens of FCCI layers. Furthermore, the method reported in this study is extendable to segmentation tasks for other materials with similar resolution, texture, and contrast characteristics, paving the way for accelerated and automated analysis in characterization analysis and beyond.

## Introduction

U-Zr based metallic fuels have been recognized as a promising nuclear fuel candidate for sodium-cooled fast reactors (SFRs)References^1^. One critical phenomenon affecting the performance of these fuels is fuel/cladding chemical interaction (FCCI), which occurs when the fuel contacts the cladding^2–4^. During irradiation, fuel constituents such as uranium (U) and plutonium (Pu), along with lanthanide (Ln) products, tend to diffuse into the cladding. Simultaneously, cladding constituents such as iron (Fe) and nickel (Ni) diffuse outward into the fuel under steady-state operating conditions^5^. This interdiffusion results in the formation of FCCI layers across the fuel periphery and the inner cladding surface^6^. The cladding-side FCCI, also known as cladding wastage, is particularly concerned as it thins the cladding wall and potentially shortens the lift-time of cladding, especially at high temperatures. Consequently, FCCI has been identified as a critical factor that limits the performance of metallic fuel under steady-state operational conditions. Understanding FCCI is essential for improving the performance and reliability of U-Zr based metallic fuels in SFRs. Enhanced knowledge in this area can lead to better fuel designs and operational strategies, ultimately contributing to the safe and efficient use of these advanced nuclear fuels.

Historically, post-irradiation examination (PIE) of fuel pins from various metallic fuel programs has been the main method to study FCCI in metallic fuels. While previous PIE studies have provided moderate understanding of FCCI, further efforts are needed to deepen this understanding and expand the FCCI database, especially for full-length fuel pins irradiated at the Fast Flux Test Facility (FFTF)^7^. This database is crucial for qualifying the U-10Zr/HT9 reference fuel system.

Recently, efforts have begun to gather FCCI data from full-length U-10Zr/HT9 fuel pins irradiated at FFTF using Scanning Electron Microscopy (SEM). SEM provides microstructural and compositional data at micron to submicron scales. As more SEM data is collected, streamlining data processes and speeding up analysis is necessary. In this study, we built a dataset with manually annotated labeling for the task of layer segmentation, suitable for public testing and study. We then employed deep learning methods to automate layer segmentation.

Deep learning (DL) based computer vision (CV) methods have recently been widely adopted for object identification in natural images. However, their application to microstructural images in material science still emerges^8^. Deep learning (DL)-based models have shown significant improvements in object detection and instance segmentation tasks, making them particularly suitable for our task of layer segmentation. Classical methods such as U-Net based architectures^9^, You Only Look Once (YOLO) series^10^, Mask Regional Convolutional Neural Network (Mask R-CNN)^11^, and the Detection Transformer (DETR)^12^ series are widely used, especially for segmentation tasks involving natural and medical objects.

**Fig. 1:**
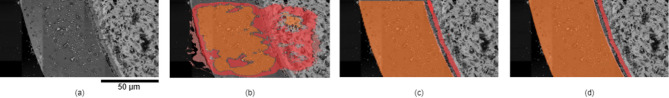
Illustration of instance segmentation of interaction layers on an example SEM image. (**a**) An input of as-collected SEM micrograph. (**b**) Segmentation results generated using a widely used method^19^. The red regions represent the near fuel interaction layer, orange areas indicate the far area of cladding contacting with fuel interaction, and the flesh color shows the interaction layer between fuel and cladding. (**c**) Results obtained using our deep learning-based model. (**d**) Manually labeled target for evaluation.

Transfer learning techniques provide certain solutions. Intelligent identification of delamination has been performed by integrating CNN-based frameworks with deep transfer learning^13,14^. Some UNet-based deep learning methods are transferred to detect ductile, brittle, and fatigue fractures in various steels and alloys^15–18^. However, accurate identification of the microscopic FCCI layers has yet to be explored. The UNet-based Resnet method^19^ is tested to as-collected SEM-based FCCI data (Fig. 1a), and the results (Fig. 1b) showed poor performance. Transfer learning offered limited applicability due to the low contrast and limited differentiability in our SEM micrographs, with some regions nearly indistinguishable from the background (Fig. 1a). It indicates that popular models with transfer learning are insufficient for FCCI layers segmentation task.

This work addresses challenges by creating a new database of as-collected SEM-based FCCI data and developing an efficient instance segmentation method for layer segmentation. We manually labeled the data with pixel-level annotations (Fig. 1d) for general segmentation of FCCI layers. The YOLOv9^20^ model, efficient in both performance and computational costs, was chosen over larger models with extensive parameters. This model uses a lightweight deep neural network architecture to preserve input information effectively. Additionally, we integrated a local-global attention module to encode the local structures of layer morphology relative to global surroundings, improving segmentation performance. The model can automatically and accurately segment the FCCI layers, as shown in Fig. 1c, using SEM micrographs. In summary, we explored deep learning methodologies to automatically analyze FCCI layer features. This approach could enable real-time, automated data analysis during data collection.

### Model development

**Fig. 2:**
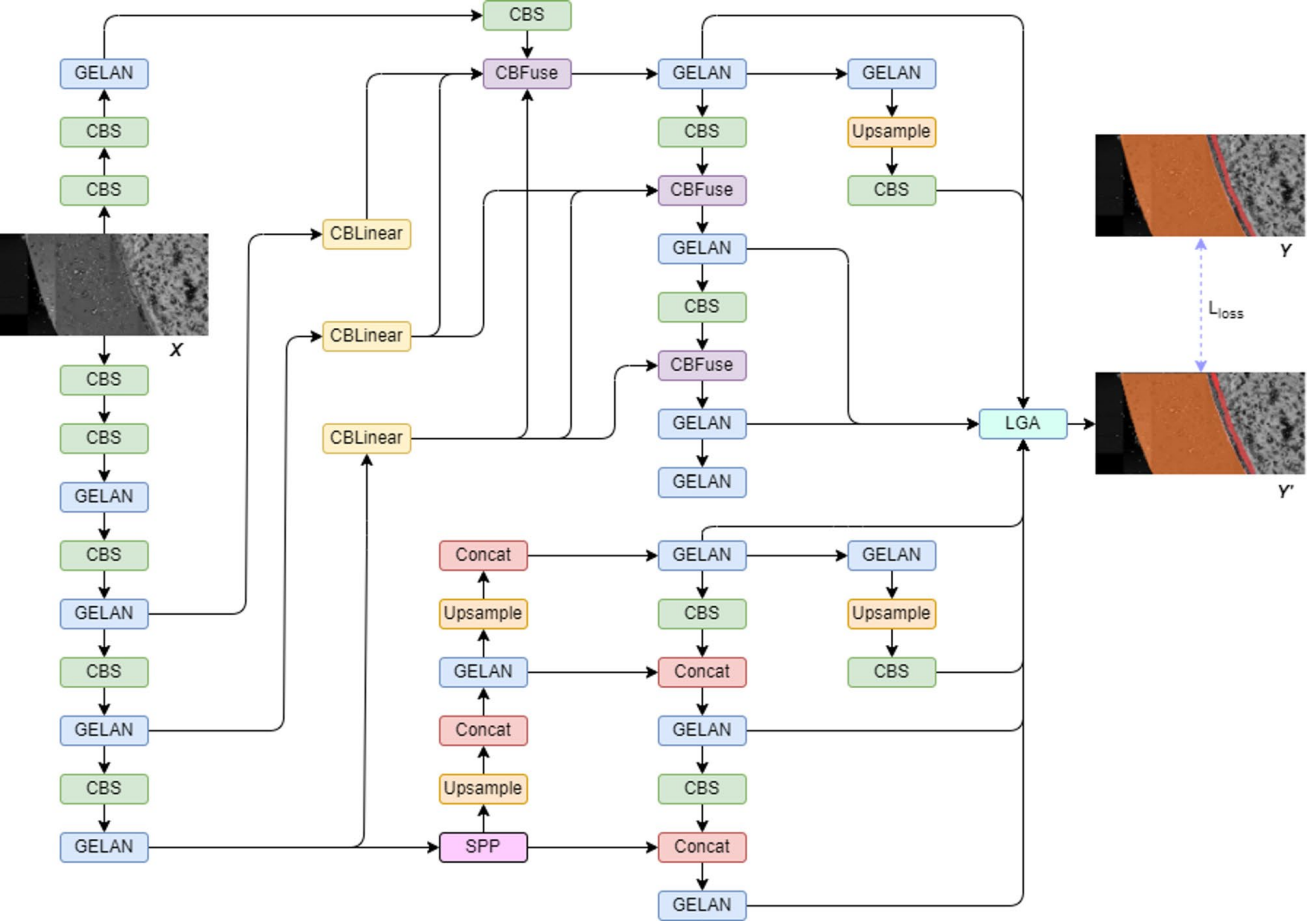
Overview of the architecture. The input SEM micrograph $\:\text{X}$ was fed into a YOLOv9-based framework for training. Each module consists of different convolutional layers and configurations, as detailed in Fig. 3. The predicted interaction layers $\:\text{Y}{\prime\:}$ were compared with the ground truth $\:\text{Y}$.

DL methods of the YOLO series represent state-of-the-art techniques for real-time object detection in natural image datasets such as Microsoft Common Objects in Context (MS COCO). These models have consistently achieved significant advancements in efficiency, accuracy, and adaptability. To solve our problem of corrosion segmentation, we developed an instance segmentation method based on YOLOv9, a recently released version of the series. This version introduced groundbreaking techniques such as Programmable Gradient Information (PGI) and the Generalized Efficient Layer Aggregation Network (GELAN). Figure 2 shows the entire architecture of the model. The micrograph input was fed into a main branch (the left part) to extract multiscale features and processed through auxiliary branch (the right part) for additional refinement. The features were then concatenated into the prediction head to obtain segmentation results. To adapt the model to our specific tasks, we replaced the original detection head with a segmentation head and adopted a local-global attention module to enhance the layer details of morphology. In the following sections, we provide a detailed explanation of the architecture of the modules, then introduce the data used in our methods, and finally outline the training and testing processes.

### Architecture

**Fig. 3:**
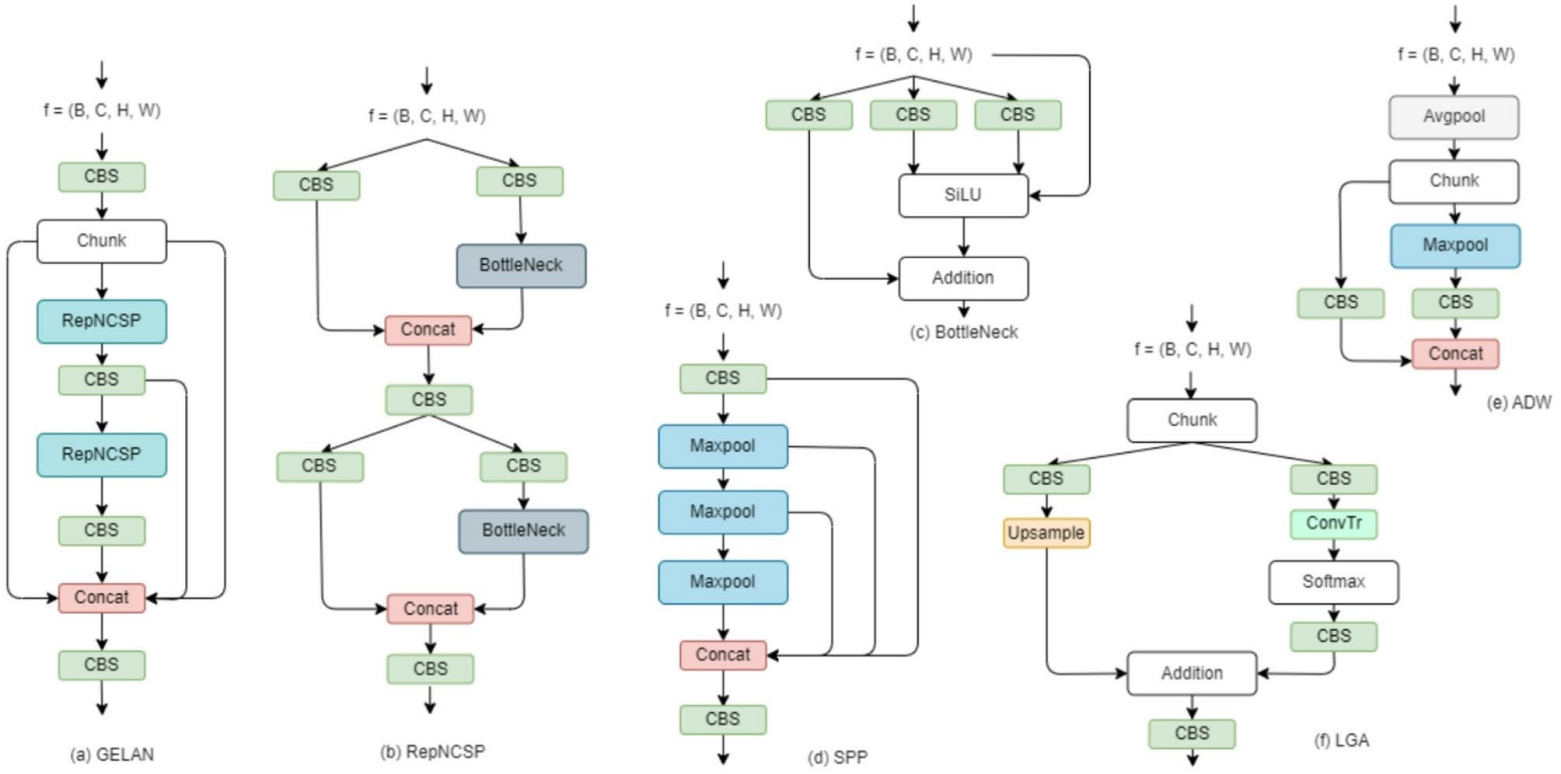
Illustration of multiple modules. (**a**) The GELAN module, which consists of CBS, CON, and RepNCSP blocks. (**b**) The configuration of the RepNCSP block. (**c**) The BottleNeck block. (**d**) The SPP block. (**e**) The ADW block. (f) The LGA block. Most blocks not shown in detail contain one single computational layer as indicated by their names. ConvTr block represents the transpose convolutional layer with normalization.

As shown in Fig. 2, the input micrograph $\:X$ was fed into the main branch. It consists of multiple modules, including the Convolution, Batch Normalization, and Swish Activation (CBS), GELAN, Average Down-sample (ADW), Spatial Pyramid Pooling (SPP), Up-sampling (UP), and Concatenation (CON) blocks. Specifically, the Upsample and Concat blocks represent up-sampling and concatenation layers, respectively. The other blocks contain multiple layers as shown in Fig. 3. The basic block CBS consists of a 2D convolutional layer equipped with a batch normalization layer and a Sigmoid Linear Unit (SiLU)^21^ activation layer. Together with layers of the average pooling, maximum pooling, and concatenation as shown in Fig. 3e, the ADW module could gradually down-sample the features to extract multi-scale features. Equipped with maximum pooling layers, the SPP block (Fig. 3d) outputs all the down-sample features. The GELAN block (Fig. 3a) consists of RepNCSP^22^ blocks (Fig. 3b) and BottleNetck blocks^23^ (Fig. 3c). The lightweight GELAN is based on gradient path planning, which can achieve better parameter utilization than the traditional depth-wise convolution. It further facilitates computational complexity, accuracy, and inference speed.

During training, the input micrograph $\:X$ was also fed into the auxiliary branch (the right part in Fig. 2), which is the reversible design of PGI to mitigate information loss. The CBLinear block was introduced to aggregate gradient information from the main branch. It consists of three 2D convolutional layers. Subsequently the CBFuse block combined the information with the auxiliary branch using one resizing and one pixel-level addition layer. The purpose of the auxiliary branch is to generate reliable gradients and maintain key characteristics within the deep representations. This ensures that features are more effective for the target task because each feature pyramid collects comprehensive information about all targets. It solves the issue of data loss for lightweight models, especially when the training data is limited. Notably, the issue of data loss often becomes severe as information propagates through deep networks. The problem arises when the convolutional network maps attributes between input and target. The loss of information results in incorrect gradient updates and consequently inaccurate predictions. According to the information theory^24^, a reversible function $\:{v}_{\tau\:}(\bullet\:)$ is the inverse transformation of function $\:{r}_{\omega\:}(\bullet\:)$ if $\:X={v}_{\tau\:}\left({r}_{\omega\:}\right(X\left)\right)$, where $\:\tau\:$ and $\:\omega\:$ are parameters of $\:v$ and $\:r$, respectively. Thus, there is no information loss with reversible functions, as represented by $\:I\left(X,X\right)=I\left(X,{v}_{\tau\:}\left({r}_{\omega\:}\right(X\left)\right)\right)$, where $\:I$ is mutual information. To mitigate information loss, the auxiliary branch was adopted to provide important information $\:I(Y,X)$, which maps data $\:X$ to the target $\:Y$ instead of relying solely on $\:I(X,\:X)$. Formally, the relationship $\:I(Y,\:X)\ge\:I(Y,\:{f}_{\theta\:}(X\left)\right)\ge\:\cdots\:\ge\:I\left(Y,\:{Y}^{{\prime\:}}\right)$ holds, where $\:{f}_{\theta\:}(\bullet\:)$ is the transformation function or model with parameters $\:\theta\:$, $\:Y$ is the target, and $\:{Y}^{{\prime\:}}$ is the predicted result. Complementary information is combined with main branch information. When the objective function was calculated with more complete information, the prediction was improved.

Here we propose an innovative approach incorporating a local-global attention module (LGA)^25^ that can be combined with the framework to guide the transfer learning of FCCI layer segmentation. The LGA module is integrated before the final prediction head. Based on fused features, the module extracted local details, enabling the accurate identification of layer ranges distinct from global backgrounds. Formally, $\:LGA\left({f}_{l},\:{f}_{g}|\gamma\:\right)=\gamma\odot{f}_{l}+{f}_{g}$ where $\:{f}_{l}$ denotes the extracted local feature maps, $\:{f}_{g}$ is the global feature maps, $\:\gamma\:$ is modulation parameter learned during training, and $\odot$ represents element-wise multiplication or Hadamard product. The LGA module synthesized multiple feature maps to increase the accuracy of prediction in detecting subtle and indiscernible FCCI layer morphologies. In the task of FCCI layer segmentation, accurately identifying layer ranges is important while the thickness of the interaction indicates the degree of fuel/cladding transition performance.

Finally, the multiscale features were concatenated and fed into the prediction head. It sequentially applied the classification and segmentation layers to predict the mask $\:Y{\prime\:}$. Specifically, the detection results were generated by the output convolutional layer on the fused features. It calculated the detection loss $\:{\mathcal{L}}_{1}$, which consisted of the Binary Cross Entropy (BCE) classification loss $\:{\mathcal{L}}_{cls}$ and the box IoU loss $\:{\mathcal{L}}_{iou}$ during the training. Formally, $\:{\mathcal{L}}_{cls}=-{\sum\:}_{i=1}^{N}{w}_{i}\:\left[{y}_{i}\text{log}\sigma\:\left({{y}_{i}}^{{\prime\:}}\right)+\left(1-{y}_{i}\right)\:\text{log}\left(1-\sigma\:\left({{y}_{i}}^{{\prime\:}}\right)\right)\right]/N$, where $\:N$ is the batch size, $\:w$ is the weight, $\:y$ is the target label, $\:{y}^{{\prime\:}}$ is the predicted category, and $\:\sigma\:$ is the sigmoid function. $\:{\mathcal{L}}_{iou}=1-{\sum\:}_{i=1}^{N}({I}_{i}/{U}_{i})/N$, where $\:{I}_{i}$ is the intersection of target and prediction boxes, $\:{U}_{i}$ is the union of them. Then, the segmentation candidates $\:Y{\prime\:}$ was predicted through the sigmoid layer. The prediction results were compared with the ground truth $\:Y$ with pixel-level BCE segmentation loss $\:{\mathcal{L}}_{2}$. Formally, the objection is $\:{\mathcal{L}}_{2}=-\sum\:_{i=1}^{HW}\left({Y}_{i}\text{log}{Y}_{i}^{{\prime\:}}+\left(1-{Y}_{i}\right)\text{log}\left(1-{Y}_{i}^{{\prime\:}}\right)\right)$, where $\:H$ is the height and $\:W$ is the width of the image. The final optimization objection is the sum of two losses: $\:\mathcal{L}={\mathcal{L}}_{1}+{\mathcal{L}}_{2}$.

In the inference stage, the features only merged information from the main branch, without features from the auxiliary branch. Instead of losses, the Non-Maximum Suppression (NMS) is used to filter out redundant results on predictions.

### Data

The model was trained on one public dataset and one newly constructed dataset. The original YOLOv9 was initialized on the public dataset, i.e., MS COCO dataset^26^, which are normally used for natural object detection and segmentation. The model developed in this study was trained on our newly constructed FCCI dataset.

MS COCO is a large-scale dataset for object detection and segmentation. In the 2017 released version, the dataset consists of 164 K natural scenes with training/validation/test split of 118 K/5K/41K. It contains 80 categories of objects, such as cars, bikes, airplanes, apples, boats, horses, kites, etc. However, it does not include categories for layers, or similar objects. The dataset was used to pre-train the model from scratch.

**Fig. 4:**
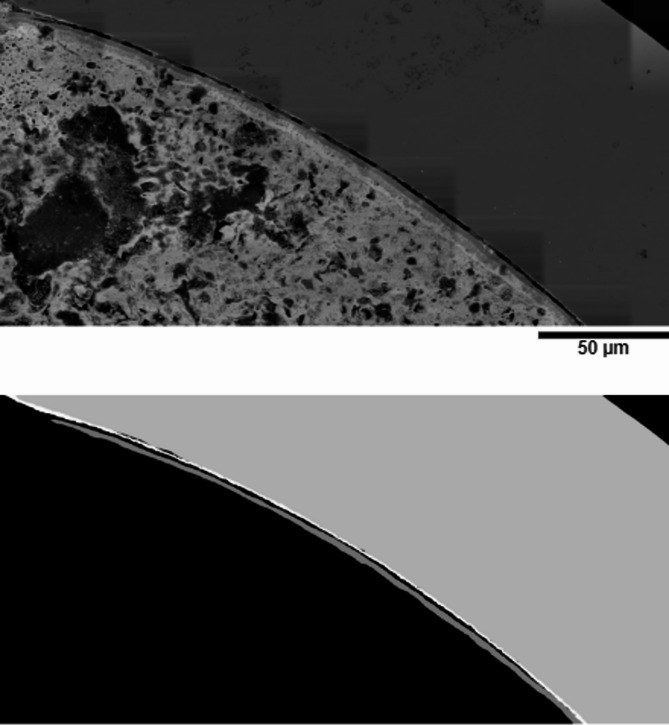
Illustration of data labeling. Top shows an example of collected SEM micrographs. Bottom displays the corresponding multi-class masks indicating the 3 layers.

Our data (as shown in Fig. 4 top) presents greater challenges in FCCI layer segmentation. Obviously, the layers, specifically the near fuel layer morphologies, show similar and tiny contrast to their surroundings, with subtle texture changes in localized regions. Additionally, some layers are very thin, and the attribute is difficult to discern. In cases where no attribute exists, the layer pattern could be easily confused with the surrounding areas. Furthermore, the boundaries of layers are often not clear. Clearly, pure transfer learning does not work for our task. The newly labeled dataset, consisting of 55 training images and 6 testing images, was constructed primarily to address these challenges and adapt to the requirements of layer segmentation. The entire dataset comprises backscattered electron images collected from the G3 Plasma Focused Ion Beam (PFIB) instrument in the Irradiated Material Characterization Laboratory (IMCL) facility at Idaho National Laboratory (INL). Three cross-section montages on the MFF2 fuel pin #192,167, which is U-10Zr fuel with HT9 cladding^27^ were investigated.

**Fig. 5:**
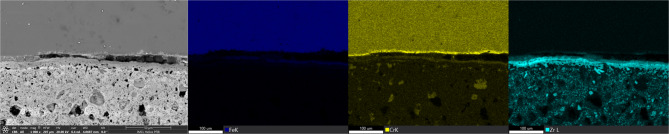
SEM EDS elemental maps used to guide the manual labeling process.

To construct the dataset from scratch, we first collected SEM micrographs from different areas on the U-10Zr. We then selected valid data and cut them into discernible scales. Specifically, a comprehensive process included identifying duplicates in different conditions, removing images with slight changes, and filtering out images with little discernible attributes. Then, we cut the images into small patches with layers, which were designed to balance the data quality. Compared with the layer near cladding, the interaction layer near fuel is too thin to identify. Finally, we manually labeled the ground truth for each micrograph. This step was, however, labor intense and expensive as the layer features were often difficult to identify due to the low contrast and diverse shapes. To improve the efficiency of this step, we adopted an interactive tool^28^ to assist in drawing masks using human vision. The manual labeling process was guided by SEM Energy Dispersive X-ray Spectroscopy (EDS) maps, which illustrate the connection between SEM features and various layers, as shown in Fig. 5. The cladding layer without FCCI regions shows high intensity on the Fe EDS map; the FCCI regions in the cladding exhibit the highest intensity on the Cr EDS map; and the FCCI region on the fuel side shows high intensity on the Zr EDS map. We also consulted the team members with FCCI expertise to guide the labeling process when the layer morphologies were complicated to delineate. We interactively annotated each FCCI image to achieve the most accurate shapes possible. We refined the annotations three times for all the micrographs. Finally, FCCI experts were again engaged to review and verify each annotation for correctness. Examples of the annotated images are shown in Fig. 4 bottom.

### Implementation

The model was implemented using Pytorch^29^. The batch size was set to 8 for training. The parameters were updated by Adam optimizer^30^. The initial learning rate was 1 × 10^−3^, and the weight decay was 5 × 10^−4^. The analysis was conducted on a single Tesla V100 graphics processing unit. The training process was conducted in two steps. First, the model was initialized with pre-trained weights on MS COCO. In the second step, the model was trained on our newly constructed FCCI layer dataset for an additional 300 epochs. In the inference stage, we used the testing data to evaluate the segmentation performance from the model. Specifically, the quantitative evaluation metrics were calculated by comparing the predicted results with the ground truth.

To enhance the diversity of the training data and improve the predicting capability of the model, the data was augmented by rotating at 0°, 90°, 180°, 270° and flipping. The augmentation was implemented by a combination of rotations with horizontal, vertical, and dual axis flips at each angle. Additionally, random cropping, brightness adjustment, and resizing were adopted. Specifically, the augmentation process included: a 50% probability of horizontal flipping, random cropping within a range from 0 to 20%, random rotations from − 15 to 15 degrees, random shearing from − 5 to 5 degrees both horizontally and vertically, random brightness adjustment within a range from − 25% to 25%, and random exposure adjustment within a range from − 25% to 25%.

## Results

This section presents the results in two parts: initial layer segmentation and improved results using the developed method. Comprehensive results and quantitative statistics are provided for detailed evaluations.

### Initial segmentation

**Fig. 6:**
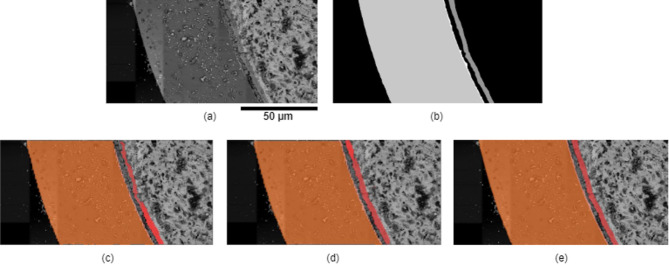
Comparison of results. (**a**) Input example. (**b**) Manually labeled ground truth. (**c**) Initial segmentation results without the proposed module. (**d**) Final segmentation results with the proposed module. (**e**) Ground truth visualized with the input SEM image.

The initial layer segmentation results were obtained from the model that was trained on the mentioned datasets but without incorporating the proposed local-global attention module. The purpose of this step was to identify the effectiveness brought by the proposed module. To facilitate visual analysis, the predicted masks were overlaid on the original SEM micrographs, enabling a direct comparison of the performance against the ground truth. As shown in Fig. 6, an example input was fed into the model, and the initial result is depicted in Fig. 6c. Compared with the ground truth, some layer regions were not captured due to the similar texture and low contrast of the image. The low-contrast attribute was a major challenge in our data, making it difficult for the model to distinguish the FCCI layers from each other and its surroundings. This in turn impacted on the final accuracy performance of the model. Even with enough data to customize the model, it was insufficient to fully address the challenges presented by our task.

### Improved segmentation

**Fig. 7:**
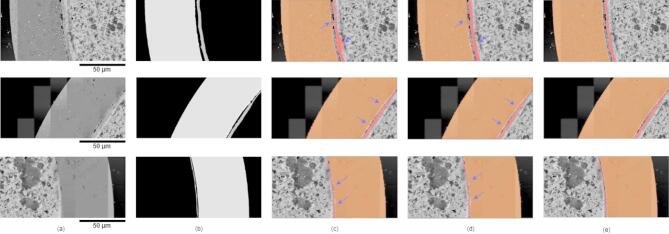
Layer segmentation results. (**a**) Input examples. (**b**) Manually labeled ground truth. (**c**) Initial layer segmentation without the local-global attention module. (**d**) Final segmentation results with proposed module. (**e**) Ground truth visualized in original SEM images. Differences between (**c**) and (**d**) are indicated with blue arrows.

To alleviate the problems in FCCI layer segmentation task and overcome the data limitations, we purposefully integrated the local-global attention module into the entire training process. With the same procedures, the results are given in Fig. 7d. Compared with the initial results illustrated in Fig. 7c, the improved result accurately identified most of missed areas with a well-defined boundary. Compared with the ground truth in Fig. 7e, the model with the developed module segmented nearly all the three layer regions in the micrograph. This demonstrates the validity of our proposed module, which can help the model to discern the local variations and improve the global segmentation of layer morphologies.

### Overall results

The refined model was applied to all SEM micrographs in the testing group, with three examples shown in Fig. 7. Due to FCCI layers with varying morphologies involved in SEM micrographs, the details are typically characterized as tiny variations. In addition to differences in lighting and contrast levels, the surrounding regions also exhibited similar features, especially near fuel areas. The ground truth for the layer morphology was labelled in Fig. 7b. For the initial segmentation results shown in Fig. 7c, most of the large layers near the cladding were segmented. However, more accurate shapes of near fuel layers were captured in the final results in Fig. 7d. Comparing Fig. 7c, d, it is evident that some layer areas were incorrectly predicted in Fig. 7c, which was obtained without the proposed local-global attention module. In contrast, the segmented layer areas shown on Fig. 7d with the proposed module closely matched the ground truth. These layer areas near the fuel are relatively smaller and display less attributes compared to the layer near the cladding. Further, some layer areas lack clear boundaries. Compared to the results in Fig. 7c, the improved results (in Fig. 7d) show more accurate shapes and continuity of the layer areas and less false positives. Despite the improved accuracy, the segmentation of some layer areas was confounded by the surrounding areas. This needs to be improved further in future studies. One typical example, shown in the second row of Fig. 7, highlights how the layer segmentation of near the fuel areas differentiates in the gradual transitions of layer thickness. While humans can hardly discern the levels of layers, the model proceeds with much subtle distinction. The middle layer (flesh color) predicted with proposed module is much smoother and continuing than that predicted without our proposed module. There are not many dents and holes in the predicted layer. The layer near the fuel (red color) predicted with proposed module is thinner by filtering out more pores between the two layers. Overall, the developed model identified most layer details and generated shapes that are highly consistent with the ground truth.

The results shown above demonstrate that our method is effective in automatically segmenting FCCI layers in SEM data. Although minor discrepancies exist compared to the ground truth, these errors mainly are localized to small regions with a high density of layer confusions. Such challenges arise due to low contrast and transitional areas, especially for the areas with fragmented intermittent and transformative features. Additionally, there were varying degrees of interactions with similar but subtly different morphologies within the same regions, as well as tiny interaction areas that may not be captured in low magnification images.

The statistics of the segmentation results were also generated and presented in Table 1. The metrics used in the experiments include precision, recall, and mean average precision (mAP) for pixel-level segmentation. Conventionally, True Positives (TP) means the number of instances where the model predictions match correctly the ground truth. True Negatives (TN) denotes the number of instances predicted by the model that do not match the ground truth. False Positives (FP) presents the number of instances from model prediction that do not match the ground truth. False Negatives (FN) is the number of instances in the ground truth that the model fails to predict. The formulas for the metrices are: $\:Precision=TP/(TP+FP)$, $\:Recall=TP/(TP+FN)$, and $\:mAP=1/N{\sum\:}_{i=1}^{N}{AP}_{i}$, where $\:AP={\int\:}_{0}^{1}P\left(R\right)dR$ is the area under the precision-recall curve $\:P\left(R\right)$, and $\:N$ is the number of images. These quantitative evaluations of the data show the robustness of our method. The metrics were calculated by considering three situations: (1) the model trained without the newly constructed dataset, (2) the model trained with the newly constructed dataset but without the texture refinement module, and (3) the model with both newly constructed dataset and the proposed local-global attention module. All models were trained in the same environment and conditions. The quantitative improvements achieved by our model are shown in Table 1. Notably, the overall accuracy of FCCI layer segmentation improved significantly from 7.3% to 92.2%. Similarly, the mAP improved from 4.4% to 90.8%. While the time cost increased from 0.4 s to 1 s per image, the difference is relatively small compared to the substantial improvement in accuracy. This trade-off highlights the efficiency of the proposed method, as the improvement in performance outweighs the marginal increase in computational time. Such enhancements make the approach practical for real-world applications where both accuracy and time efficiency are critical. Moreover, we have evaluated the model’s performance on individual FCCI layers as shown in Table 2. Each layer was evaluated using 6 images and 6 instances. For the layer near the fuel, the new proposed model demonstrated a high precision of average 94.0%, indicating that most of the identified pixels of this layer were correct. The recall was very high at 100%, suggesting that the model predicted few false negative or missed instances. The average precision (mAP) was high at 99.5%, reflecting that on average most detected instances were highly accurate. In the layer between the fuel and cladding, a precision of 82.7% and a recall of 83.3% were achieved. The mAP for this layer was 81.3%, indicating overall strong performance and reliability in detecting this layer. Noted that this layer is extremely thin between the other two layers, and it has noisy surroundings of the layer boundaries. The mAP was 91.5% for the layer near cladding, demonstrating that the model was effective in identifying these instances.

**Table 1:** Quantitative results of testing samples.

Experiments	Images (#)	Instances (#)	Precision (%)	Recall (%)	mAP (%)	Time (seconds)
1	6	18	7.3	18.3	4.4	0.1
2	6	18	87.5	81.5	85.6	0.4
3	6	18	92.2	88.7	90.8	1.0

The metrics were presented as percentage values with numerical counts denoted by “#” and time represented in seconds. Index number 1, 2, and 3 indicate results obtained without our dataset, without the proposed local-global attention module, and with the proposed module, respectively.

**Table 2:** Quantitative results of testing samples for each layer.

Layers	Color in Fig.7b	Images (#)	Instances (#)	Precision (%)	Recall (%)	mAP (%)
FCCI layer in the fuel	Light gray	6	6	94.0	100.0	99.5
FCCI layer in the cladding	white	6	6	82.7	83.3	81.3
Cladding matrix	Dark gray	6	6	100.0	82.6	91.5

The metrics were presented as percentage values or numerical counts denoted by “#”.

The quantitative comparison of different existing models with transfer learning is shown in Table 3. We collected several representative models used for microstructural segmentation. Each method is transferred to our built dataset and tested on the same images. Our method consistently outperforms the popular methods.

**Table 3:** Quantitative comparison of popular existing methods.

Methods	Images (#)	Instances (#)	Precision (%)	Recall (%)	mAP (%)	Time (seconds)
FPN EfficientNet^31^	6	18	65.6	68.9	66.1	0.5
FPN InceptionResnet^32^	6	18	54.3	55.9	52.9	0.4
FPN Resnet^33^	6	18	66.5	61.7	60.9	0.3
VGG Resnet^34^	6	18	64.2	65.8	64.6	0.6
Linknet Resnet^35^	6	18	63.5	57.3	57.0	0.4
Unet Resnet^36^	6	18	63.3	61.3	59.1	0.3
SegNet^37^	6	18	51.0	50.4	49.0	0.3
Ours	6	18	92.2	88.7	90.8	1.0

The metrics were presented as percentage values with numerical counts denoted by “#”. Each method is trained and tested on our dataset.

To ensure the robustness of the model, we conduct cross validation on the proposed method in Table 4. Experiment 1 corresponds to the final results (reported in Tables 1 and 3) and is included here for comparison. Experiments 2 to 6 are five additional trials using different random splits of the dataset, each with 55 images for training and 6 images for testing. Each image might have multiple instances with broken layers. Although the number of images remain constant, the specific images differ across splits, leading to variations in the total number of layer instances. When difficult samples are randomly selected into training sets and easy samples are divided into testing sets, the performance is increased in many metrics. As a result, some fluctuations are observed in precision, recall, and mAP metrics. Nevertheless, the results consistently demonstrate the validity of the proposed method.

**Table 4:** Cross validation of the proposed method.

Experiments	Images (#)	Instances (#)	Precision (%)	Recall (%)	mAP (%)	Time (seconds)
1	6	18	92.2	88.7	90.8	1.0
2	6	23	96.3	75.9	86.6	1.0
3	6	20	96.5	95.8	97.1	1.0
4	6	23	92.7	93.1	93.6	1.0
5	6	21	95.5	78.8	92.4	1.0
6	6	19	96.2	89.5	94.3	1.0
Average	6	21	94.9	87.0	92.5	1.0

The metrics are presented as percentage values with numerical counts denoted by “#” and time represented in seconds. Indices 1 through 6 indicate results obtained from randomly splitting the training and testing sets across six independent runs.

## Discussion

In this study, we addressed the significant challenges presented by as-collected SEM micrographs, characterized by their dense, complex, and highly interconnected layer morphologies with low contrast. Traditional manual methods, while promising for high-quality microscopy data, e.g., SEM and transmission electron microscopy (TEM) data, have proven inadequate for our specific SEM micrographs. Popular models have also performed poorly in this context.

To overcome these challenges, we developed a DL-based model incorporating a local-global attention module specifically designed for this task. Our model is trained end-to-end in existing, as-collected data, enabling direct segmentation of layer instances without the need for additional preprocessing steps. This approach contrasts with the traditional step-by-step manual processing methods.

Our method offers significant improvements in efficiency and scalability by generating pixel-level segmentation results for new SEM data within seconds. This advancement allows for more comprehensive analysis, such as combining segmentation predictions to provide a detailed view of FCCI morphologies with high accuracy and efficiency. In contrast, relying solely on cross-section data can lead to missing regions and inaccurate predictions due to thin layers near the fuel. Such an approach would also demand more model parameters, handling modules, labeled data, prediction time, and computing resources, making it impractical for real-world applications. Our method strikes a balance between accuracy and efficiency for both local and global FCCI analysis.

However, we identified some limitations in our proposed method. One major challenge is the lack of sufficient training data specific to our target task, which limits the model’s ability to learn from changing domains and feature variations. This issue is common in materials science, where obtaining adequate training data with manual labeling is often difficult. We addressed this by developing a local-global attention module that focuses on continuity and boundary problems in layer interactions. In future work, we aim to further reduce the domain gap impact on our model’s performance with SEM data, enhancing the efficiency and accuracy of FCCI layer segmentation and contributing to advancements in material degradation research.

**Fig. 8:**
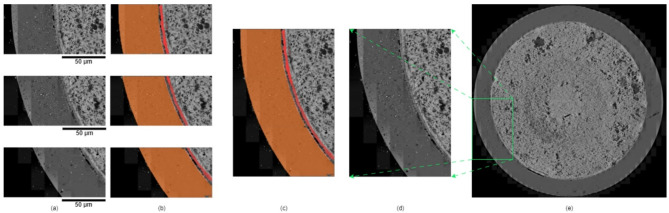
Interaction layer segmentation applications. (**a**) Input examples. (**b**) Prediction results. (**c**) Combined results. (**d**) Combined SEM micrograph. (**e**) Overall view of a HT9/U-10Zr fuel cross section.

Another limitation is the potential for human bias and errors during manual labeling of SEM data, especially on the boundaries between different layers. Despite using a tool model and consulting material experts for verification, variations due to image resolution and human visual perception persist. Standardizing the procedure for identifying and labeling FCCI layers will improve consistency and help avoid inconsistencies in model development, preventing non-robust performance in layer segmentation. This improvement is particularly important for SEM data containing various FCCI layers and interconnected morphologies with subtle differences compared to surrounding areas (Fig. 8).

## Conclusions

This work introduces a deep learning-based end-to-end method for automatically segmenting FCCI layers in SEM micrographs. The developed model can directly perform layer segmentation on as-collected SEM data without requiring any manual preprocessing, making it suitable for real-time applications. The method has two main contributions: the creation and development of an FCCI layer segmentation dataset specifically for this task, which provides a valuable resource that supports research advancements in material science and nuclear fuel studies; and an end-to-end DL-based model integrated with a local-global attention module, which significantly improves the performance of layer segmentation in SEM micrographs by comparing with state-of-the-art DL models. This approach effectively addresses the challenges posed by transformative variations in low-contrast SEM micrographs. The results demonstrate the robustness of the developed model in handling FCCI layer segmentation tasks for SEM micrographs. Additionally, the model can be extended to general layer features segmentation tasks. This advancement has the potential to accelerate automated processes and further propel research in material science.

## Data Availability

Data will be made available on reasonable request. Contact Liang Zhao at Liang.Zhao@inl.gov and Yachun Wang at yachun.wang@inl.gov.

## References

[1] Walters, L. C., Seidel, B. R. & Kittel, J. H. Performance of metallic fuels and blankets in Liquid-Metal fast breeder reactors. *Nucl. Technol.* **65** (2), 179–231. 10.13182/NT84-A33408 (1984).

[2] Hofman, G. L., Walters, L. C. & Bauer, T. H. Metallic fast reactor fuels. *Prog. Nucl. Energy*. **31** (1-2), 83–110 (1997).

[3] Keiser, D. Jr. Fuel cladding chemical interaction in metallic sodium fast reactor fuels: A historical perspective. *J. Nucl. Mater.* **514**, 393–398 (2019).

[4] Carmack, W. J. et al. Metallic fuels for advanced reactors. *J. Nucl. Mater.* **392** (2), 139–150 (2009).

[5] Wachs, D. M., Luca, C. & Douglas, P. Behavior of metallic fast reactor fuels during an overpower transient. *J. Nucl. Mater.* **557**, 153304 (2021).

[6] Di, L. et al. Microstructure and phase evolution in the U-10Zr fuel investigated by in situ TEM heating experiments. *J. Nucl. Mater.* **583**, 154475 (2023).

[7] Pitner, A. L. & Baker, R. B. Metal fuel test program in the FFTF. *J. Nucl. Mater.* **204**, 124–130. 10.1016/0022-3115(93)90208-G (1993).

[8] Holm, E. A. et al. Overview: computer vision and machine learning for microstructural characterization and analysis. *Metall. Mater. Trans. A*. **51**, 5985–5999 (2020).

[9] Ronneberger, O., Fischer, P. & Brox, T. U-net: Convolutional networks for biomedical image segmentation. In *Medical image computing and computer-assisted intervention.* pp 234–241. (2015).

[10] Redmon, J. You only look once: Unified, real-time object detection. In *Proceedings of the IEEE conference on computer vision and pattern recognition.* (2016).

[11] He, K., Gkioxari, G., Dollár, P. & Girshick, R. Mask r-cnn. In *Proceedings of the IEEE international conference on computer vision.* pp 2961–2969. (2017).

[12] Carion, N. et al. End-to-end object detection with transformers. In *European conference on computer vision.* pp 213–229. (2020).

[13] Azad, M., Muzammil, P., Kumar & Heung Soo Kim. Delamination detection in CFRP laminates using deep transfer learning with limited experimental data. *J. Mater. Res. Technol.* **29**, 3024–3035 (2024).

[14] Azad, M., Muzammil & Heung Soo Kim. Hybrid deep convolutional networks for the autonomous damage diagnosis of laminated composite structures. *Compos. Struct.* **329**, 117792 (2024).

[15] Avilés-Cruz, C. et al. A new machine learning-based evaluation of ductile fracture. *Eng. Fract. Mech.* **302**, 110072 (2024).

[16] Tang, K. et al. Deep learning-based semantic segmentation for morphological fractography. *Eng. Fract. Mech.* **303**, 110149 (2024).

[17] Kaya, G. U. Development of hybrid optical sensor based on deep learning to detect and classify the micro-size defects in printed circuit board. *Measurement* **206**, 112247 (2023).

[18] Zhao, L. et al. Quantification of line dislocations in FFTF irradiated HT9 cladding by deep learning method. *Mater. Charac.* **96**, 115322 (2025).

[19] Wang, H. et al. A fine pore-preserved deep neural network for porosity analytics of a high burnup U-10Zr metallic fuel. *Sci. Rep.* **13** (1), 22274 (2023). 38097710 10.1038/s41598-023-48800-3 PMC10721912

[20] Wang, C. Y., Yeh, I. H. & Mark Liao, H. Y. Yolov9: Learning what you want to learn using programmable gradient information. In *European conference on computer vision*. (2024).

[21] Elfwing, S., Uchibe, E. & Doya, K. Sigmoid-weighted linear units for neural network function approximation in reinforcement learning. *Neural Netw.* **107**, 3–11 (2018). 29395652 10.1016/j.neunet.2017.12.012

[22] Wang, C. Y. et al. CSPNet: A new backbone that can enhance learning capability of CNN. *Proceedings of the IEEE/CVF conference on computer vision and pattern recognition workshops*. (2020).

[23] He, K. et al. Deep residual learning for image recognition. *Proceedings of the IEEE conference on computer vision and pattern recognition*. (2016).

[24] Tishby, N. & Zaslavsky, N. Deep learning and the information bottleneck principle. In *2015 ieee information theory workshop (itw)* pp. 1–5. (2015).

[25] Zhao, L. et al. Background-insensitive scene text recognition with text semantic segmentation. In *European Conference on Computer Vision.* pp. 163–182. (2022).

[26] Microsoft Common Objects in. Context (MS COCO) dataset, URL: https://cocodataset.org/

[27] Pitner, A. L. & Baker, R. B. Metal fuel test program in the FFTF. *J. Nucl. Mater.* **204**, 124–130 (1993).

[28] Sun, S., Xian, M., Xu, F., Capriotti, L. & Yao, T. Cfr-icl: Cascade-forward refinement with iterative click loss for interactive image segmentation. In *Proceedings of the AAAI conference on artificial intelligence.* Vol. 38, No. 5, pp. 5017–5024. (2024).

[29] Imambi, S., Prakash, K. B. & Kanagachidambaresan, G. R. PyTorch. *Programming TensorFlow: Solut. Edge Comput. Applications* pp.87–104. (2021).

[30] Kingma, D. P. Adam: A method for stochastic optimization. *arXiv preprint arXiv:1412.6980* (2014).

[31] Thai-Nghe, N. & Van Kiet, V., Nguyen, H.-H. Brain Tumor Segmentation with FPN-Based EfficientNet and XAI. *Asian Conference on Intelligent Information and Database Systems*. Singapore: Springer Nature Singapore, (2024).

[32] Yadav, A. & Kumar, E. Object detection on Real-Time video with FPN and modified mask RCNN based on Inception-ResNetV2. *Wireless Pers. Commun.* **138** (4), 2065–2090 (2024).

[33] Li, Z. et al. Detnet: A backbone network for object detection. *arXiv preprint arXiv:1804.06215* (2018).

[34] Sheiati, S. et al. Cementitious phase quantification using deep learning. *Cem. Concr. Res.* **172**, 107231 (2023).

[35] Zhang, R. et al. Comparison of backbones for semantic segmentation network. *Journal of Physics: Conference Series*. Vol. 1544. No. 1. IOP Publishing, (2020).

[36] Saha, A., Zhang, Y. D. & Suresh Chandra Satapathy. Brain tumour segmentation with a muti-pathway ResNet based UNet. *J. Grid Comput.* **19** (4), 43 (2021).

[37] Sheiati, S., Behboodi, S. & Navid Ranjbar. Segmentation of backscattered electron images of geopolymers using convolutional autoencoder network. *Expert Syst. Appl.* **206**, 117846 (2022).

